# The Criteria to Confirm the Role of Epstein-Barr Virus in Nasopharyngeal Carcinoma Initiation

**DOI:** 10.3390/ijms131013737

**Published:** 2012-10-23

**Authors:** Ai-Di Gu, Mu-Sheng Zeng, Chao-Nan Qian

**Affiliations:** 1 State Key Laboratory of Oncology in South China, Sun Yat-sen University Cancer Center, 651 Dongfeng East Road, Guangzhou 510060, China; E-Mails: topgad@yahoo.com (A.-D.G.); zengmsh@sysucc.org.cn (M.-S.Z.); 2 Lineberger Comprehensive Cancer Center, Department of Microbiology and Immunology, University of North Carolina, Chapel Hill, NC 27599, USA; 3 Laboratory of Cancer and Developmental Cell Biology, Van Andel Research Institute, 333 Bostwick Ave. NE, Grand Rapids, MI 49503, USA

**Keywords:** Epstein-Barr virus, nasopharyngeal carcinoma, tumorigenesis, infection

## Abstract

Epstein-Barr virus (EBV) is associated with nasopharyngeal carcinoma (NPC), but it remains obscure whether EBV is a viral cause of, or only an accompaniment of, NPC. We will discuss the accumulated evidence pointing to the relationship between EBV infection and NPC initiation from epidemiologic, pathogenic, molecular oncogenic, and experimental animal studies. We believe that convincing evidence from these perspectives must be provided before we can ascertain the causal role of EBV infection in NPC. Specifically, (1) epidemiological studies should reveal EBV infection as a risk factor; (2) the introduction of EBV into an animal model should produce NPC; (3) in the animal model NPC, the main molecular event(s) or the involved signaling pathway(s) should be identical to that in human NPC; and (4) finally and most importantly, prevention of EBV infection or clearance of EBV from infected individuals must be able to reduce the incidence rate of NPC.

## 1. Introduction

The viral cause of an infectious disease can be established by fulfilling Koch’s postulates as modified by Rivers [1], for which six criteria are required: universal presence of virus in every diseased host, isolation from hosts, cultivation in pure culture, production of a comparable disease by injecting the virus into a suitable recipient, re-isolation of the virus, and detection of a specific immune response to the virus. In 2003, when the new human infectious disease SARS (severe acute respiratory syndrome) broke out, these criteria helped to establish the causal relationship between SARS and SARS-associated coronavirus [2]. However, unlike infectious diseases, cancer occurrence is a complex and chronic process, and the association and causative relationship between virus and carcinogenesis was debated for a long time until viruses were verified in some cancers.

Epstein-Barr virus (EBV) was the first virus detected within human cancer cells. In 1964, Epstein’s group discerned virus-like particles by electron microscopy in a cell line derived from Burkitt’s lymphoma [3]. During the following 20 years, significant discoveries helped to establish the relationship between viruses and tumors, including the recovery of hepatitis B virus (HBV) particles in the serum of patients with hepatitis [4]; the isolation of the human T-cell leukemia virus (HTLV-1) from lymphocytes of a patient with cutaneous T-cell lymphoma [5]; and identification of human herpesvirus 8 as the cause of Kaposi’s sarcoma [6]. However, the role of EBV in NPC initiation is far more difficult to be clarified.

EBV is a member of the herpesvirus family and infects more than 90% of the world’s population [7]. Primary infection usually occurs in childhood and is asymptomatic in developing countries [8]. In western countries, EBV infection may be delayed until adolescence, usually with the occurrence of infectious mononucleosis [9]. EBV can exist in the human host without serious consequences for a lifetime. Nasopharyngeal carcinoma (NPC) is considered to be associated with EBV infection [10], but whether EBV plays a causal role in NPC or is only associated with its development remains controversial. In this article, we will discuss the relationship between EBV infection and NPC initiation.

## 2. Inconsistent Epidemiologic Evidence for the Etiological Role of EBV

Epidemiology studies provide the primary and essential evidence for the relationship between diseases and their causal agents. The initial link of EBV infection to NPC was discovered by serological analysis: antibody titers against EBV were higher in the sera of NPC patients than in sera from patients with Burkitt’s lymphoma [11]. Later it was found that NPC usually exhibits a high EBV serological profile wherever it occurs, whether in endemic or non-endemic areas [12,13]. Most NPC patients have high immunoglobulin A and/or IgG levels against various EBV antigens, including viral capsid antigen (VCA), diffused early antigen (EA-D), viral nuclear antigen 1 (EBNA1), glycoprotein 78 (gp78), and the transcription activators Zta and Rta [12,14,15]. Elevated EBV antibody titers have been reported to precede the development of detectable NPC by several years [16], strengthening the impression that EBV might be the etiological factor of NPC.

Even though EBV infection is ubiquitous in humans, the incidence of NPC varies with geographic area. NPC is rare in many parts of the world, including Europe and North America. A moderate incidence is found in some African and Mediterranean populations, in Inuits from Greenland and Alaska, and in Malays from Singapore and Malaysia. The highest incidence occurs in the people of southern China and Southeast Asia, particularly in the Cantonese [17]. While emigrants from southern China have a lower incidence, they still retain a higher risk of NPC than western populations [18]. Also, the risk of NPC among Caucasians born in China or southeast Asia is higher relative to that of western-born Caucasians [19]. Thus, the fact of universal EBV infection but specific endemic or racial NPC distribution suggests that nonviral factors such as environmental carcinogens or genetics may play more important roles in NPC initiation than EBV infection.

Familial clustering of NPC, which is often observed, is considered as a good model for studying NPC etiology. If EBV is a causal factor, those at high risk for NPC should have higher EBV antibody reactivities than healthy controls in the same population. Although one study showed that unaffected individuals from high-risk NPC family had higher anti-EBV antibody titres [20], our study did not find any difference in EBV serology between the unaffected family members and healthy control populations [21]. The reason for this discrepancy remains unknown, but it suggests that EBV might not be the initial factor for NPC onset. Recently, one study based on epidemiological and *in vitro* experimental data showed that smoking could result in EBV activation [22]. It is therefore believed that some other non-viral factors (e.g., genetic factor) are required for host cell carcinogenesis accompanied by EBV activation. Genome-wide linkage scans have been run on distinct series of families from south China, and different predisposition loci were found [23,24]. Although these data are not consistent, susceptibility genes or a common lifestyle might contribute to NPC given the strong familial aggregation.

## 3. Accumulated Pathological Evidence does not Support the Hypothesis of EBV Causing NPC

The idea of EBV involvement in NPC initiation comes in part from the observation that NPC tissues contain monoclonal viral episomes with identical terminus numbers [25]. Upon entry into the cell, the linear EBV DNA circularizes through its terminal repeats to form the intracellular episome and for each circularization event, the fused termini are of a unique length [26]. Unlike the length heterogeneity in a cell population at primary infection, identical terminal repeat values of EBV DNA are found in NPC samples [25]. From clonality data, it was hypothesized that NPC tumors represent the outgrowth of a single infected progenitor at the time of initiation [10]. However, *in vitro* experiments of EBV infection into established epithelial cell lines have shown that NPC cell lines are initially polyclonal, but this is rapidly followed by the predominance of a clonal pattern [27,28]. Thus, EBV clonality may be a consequence of a selective growth advantage displayed by specific viral episomes [27], arguing against the hypothesis that EBV is present prior to the clonal expansion of malignant cells. Furthermore, although most of samples of invasive NPC or NPC *in situ* are positive when examined by *in situ* hybridization for EBV-encoded RNA (EBER) [29], EBV RNA-negative cells are observed in every NPC biopsy specimen [30]. Only a small fraction of NPC cells contain the virus in the EBV-positive cell lines, and several NPC cell lines are even EBV-free [30,31].

Relative to B lymphocytes, epithelial cells do not express the C3d/EBV receptor, which makes a difference for EBV infection [32]. EBV can infect NPC cells through the contact between EBV-IgA and the polymeric immunoglobulin receptor (PIGR) [31]. Untransformed squamous metaplastic epithelial cells in the nasopharyngeal mucosa are PIGR-negative, so EBV cannot infect those cells [31], and EBV infection of normal nasopharyngeal cells is rare. EBV was not detected in normal nasopharyngeal biopsies from individuals at high risk of developing NPC, nor in normal mucosa adjacent to EBV-positive NPC [33]. Yet, PIGR protein is expressed in some tumor cells and salivary gland epithelial cells [31], which might explain why the EBV genome is less frequently detected in untransformed nasopharyngeal epithelial cells [34,35].

Among the nasopharyngeal biopsy tissues at various neoplastic stages we analyzed by PCR, 145 of 149 cases of invasive NPC and 2 of 4 cases of *in situ* NPC were positive for EBV DNase, but only 2 of 202 healthy subjects who had elevated EBV antibody levels were DNase-positive [36]. Together with the long latency between primary EBV infection and NPC development, these results suggest that EBV infection is not the first event during NPC pathogenesis.

In one study, 22 of 24 specimens of nasopharyngeal lymphoid hyperplasia contained EBV DNA, but no NPC was found among the 22 individuals after five years of follow-up [37]. Considering the losses of chromosome 3p and 9p in normal nasopharyngeal mucosa and in low-grade dysplastic lesions from individuals from high-risk NPC regions [38,39], it is possible that genetically abnormal nasopharyngeal epithelium causes a predisposition to EBV infection originating from adjacent lymphoid tissues and circulating B-cells [10]. As an example, if NPC cell lines were immortalized by overexpression of the cellular oncogene *Bmi-1*, EBV could infect such cells at a higher efficiency, suggesting that host oncogenes could favor EBV infection [40].

## 4. EBV Might Only Assist in Promoting NPC Progression

Studies of oncogenic mechanisms in cell lines are important for verifying information derived from epidemiologic observations. *LMP1* is considered as the EBV oncogene because it can transform rodent fibroblast cell lines such as Rat-1 [41]. LMP1 produces a loss of contact inhibition in Rat-1 cells and causes anchorage-independent growth in both Rat-1 and BALB/c 3T3 cells, so they clone with high efficiency *in vitro* [42]. Rat-1 cells transformed by LMP1 are tumorigenic in nude mice, while cells without LMP1 are not [41]. The expression of LMP1 also has significant effects on epithelial cell growth, inducing epidermal hyperplasia when expressed in the skin of transgenic mice [43]. However, LMP1 does not transform epithelial cells efficiently: it is detected only in 50% of NPC cases [44]. Some evidence indicates that LMP1-positive NPCs are more aggressive than LMP1-negative tumors [44,45], and there are recent reports that LMP1 and LMP2A can induce NPC stem-like cancer cells and then cause high tumorigenicity and rapid cell proliferation [46,47]. Thus, LMP1 might contribute to NPC progression by activating various signal pathways and then regulating the expression of the host genes that encode proteins involved in tumor progression and invasion, such as p16, cyclin D1, VEGF, and MMP9 and some pro-inflammation cytokines [48–50].

Although EBV can infect and transform B lymphocytes efficiently *in vitro*, epithelial infection is much less efficient [51]. The transformation of normal human epithelial cells by EBV depends on the presence of phorbol esters, which are produced by plants, and particularly by plants used for herbal medicines; such plants grow within the geographic area of NPC prevalence in South China [52]. The establishment of a cell model for EBV infection of epithelial cells that replicates the *in vivo* situation would provide important insights into the relationship of EBV and NPC.

## 5. Lack of a Convincing NPC Animal Model Induced by EBV Infection

Animal experiments can provide direct information on the relationship between carcinogenic agents and carcinomas: successful examples include Mongolian gerbils infected with *H. pylori* for studying gastric carcinoma [53] and HBV transgenic mice for studying liver cancer [54]. Appropriate animal models play an important role in the evaluation of infectious agents in carcinogenesis.

EBV is a human virus with a high degree of species specificity, and the study of this virus *in vivo* is difficult. There are two strategies for building animal models of EBV or EBV-like infection that could partly reproduce the cell/virus relationships of either symptomatic primary infection or human EBV-associated carcinomas: one is to challenge the animals with EBV, and the other is to study the virus/host relationship using the host’s resident γ-herpesviruses.

Inoculation of severe combined immunodeficient (SCID) mice with EBV-infected B cells can result in viral loads and the development of malignant lymphomas [55]. But in this model system, the host cells have never been infected by EBV, so this model is inappropriate for testing the initiation of NPC by EBV infection. A humanized mouse model for EBV infection has been established. In this model, human T, B and natural killer cells were reconstituted and EBV could infect and grow in the B cell and result in B cell lymphoproliferative disorder [56]. Whether this model could be used to study EBV-NPC relation warrants further investigation.

Primates are similar to humans, and three species of New World monkeys can be experimentally infected by EBV. For example, after inoculation with EBV at high titer, the cotton tamarin, the cottontop marmoset, and the owl monkey can develop an ill-defined infectious mononucleosis-like syndrome or multiple lymphomas [57,58]. However, these species are generally challenged by the intraperitoneal route (rather than the oral), meaning that the virus/cell relationship is established differently than that on human mucosal surfaces. Furthermore, these primate models can not establish a persistent EBV infection, and some of them have cross-species virus infection, suggesting they cannot precisely mimic the human response to EBV infection [59].

Both murine γ-herpesvirus 68 (γHV68) and rhesus lymphocryptovirus (LCV) contain genetic sequences and immune controls similar to those in human EBV, so they have been used to model persistent B-cell infection and B-cell lymphomagenesis [60,61]. LCV epithelial cell infection of the esophagus and/or tongue was observed in immunosuppressed rhesus macaques, similar to EBV-induced epithelial cell abnormalities in AIDS [62]. However, there are no reports of LCV infection that have resulted in nasopharyngeal malignancy.

## 6. Proposed Criteria to Confirm the Etiological Role of EBV in NPC

To establish an etiological factor for a certain malignancy, in particularly, the etiological role of EBV in NPC initiation, we believe the following criteria modified from Koch’s postulates should be fulfilled.

1. Epidemiological study should provide evidence of the proposed factor being an independent risk factor for the cancer’s incidence;
2. Introduction of the proposed factor into animal models should produce the malignancy, mirroring the human disease;
3. In the animal-model disease, the main molecular events or the main signaling pathway(s) involved should be identical to those of the human malignancy;
4. Finally and most importantly, preventing the proposed factor from entering a susceptible population, or clearance of the proposed factor by treatment (by vaccination in the case of EBV) in high-risk populations, must significantly reduce the incidence rate of the malignancy.

Vaccines are the most effective and economical preventive approach against viral infections, and vaccines against cancer viruses have the potential of reducing the cancer rate. Successes in the development of the HBV and HPV vaccines have demonstrated this concept. The HBV vaccine has been used for about 20 years to prevent viral transmission to newborns and the resulting life-long infections [63]. The effectiveness of the HBV vaccine in reducing liver cancer will be clear 20 years from now. HPV vaccines provide more than 90% protection against persistent HPV infection for up to five years after vaccination [64].

In order to clarify the etiological role of EBV in NPC, we believe the most convincing evidence should be the decline of the NPC incidence rate in endemic areas by prevention of EBV infection. In the 1980s, it was first reported that the immune response to purified native or recombinant gp350 (previously termed gp340) EBV protein could protect against EBV-induced lymphoma in cottontop tamarins [65]. Later, a vaccinia-delivered gp350 vaccine was reported to protect infants from EBV infection in a clinical trial of 16 months [66]. In recent years, EBV vaccines containing different antigens have been developed and tested in phase I/II clinical trials [67], but no vaccine has been taken to advanced-stage trials. Although the recombinant gp350 vaccine could reduce 78% incidence of IM, it could not prevent asymptomatic EBV infection [67]. More importantly, no evidence thus far shows that EBV vaccines are effective in protecting animal models from NPC initiation.

## 7. Conclusions

Tumor initiation is usually a complex, multistep process involving environmental, biological, and genetic factors. Tumor viruses may play important roles in carcinogenesis; they might also contribute toward uncovering the cell growth pathways of cancer [68].

Although the clinical serological data suggest an association of EBV with NPC, the evidence from mechanistic studies and animal bioassays for the role of EBV infection in NPC occurrence is weaker than that for the role of EBV in lymphomas. Furthermore, the fact of universal EBV infection and the marked geographic and racial distributions of high-incidence NPC suggest that other co-factors may play more important roles in NPC initiation. Future studies to fulfill our proposed criteria are needed to clarify the role of EBV infection in NPC initiation, which would not only solve some of the mysteries of EBV biology, but also provide benefits in NPC prevention and treatment.
